# Single dose of intravenous ferric carboxymaltose infusion versus multiple fractionated doses of intravenous iron sucrose in the treatment of postoperative anaemia in colorectal cancer patients: study protocol for a randomised controlled trial

**DOI:** 10.1186/s13063-018-3125-2

**Published:** 2019-01-07

**Authors:** María Jesús Laso-Morales, Roser Vives, Andrea Vallejo-Tarrat, Novella Caló, Anna Valle-Beltran, Caridad Pontes

**Affiliations:** 1 Department of Anaesthesiology. Parc Taulí Hospital Universitari, Institut d’Investigació i Innovació Parc Taulí I3PT, Universitat Autònoma de Barcelona, Parc Taulí 1, 08208 Sabadell, Spain; 2 Clinical Pharmacology Unit, Department of Pharmacy. Parc Taulí Hospital Universitari, Institut d’Investigació i Innovació Parc Taulí I3PT, Universitat Autònoma de Barcelona, Parc Taulí 1, 08208 Sabadell, Spain; 3 Departament de Farmacologia, de Terapèutica i de Toxicologia, Unitat Docent Parc Taulí, Universitat Autònoma de Barcelona, C/Parc Taulí, 1, 08208 Sabadell, Spain

**Keywords:** Colorectal cancer, Postoperative anaemia, Intravenous iron

## Abstract

**Background:**

Patients with colorectal cancer (CRC) often present with associated anaemia which is usually present at the time of diagnosis and is aggravated during the postoperative period due to blood loss during the surgery process. Several guidelines advocate for the treatment of postoperative anaemia in these patients in order to prevent complications and allogeneic blood transfusions. However, there are no publications to shed light on the effectiveness of intravenous iron (IVI) administration after CRC surgery and the optimal dose and regimen. We have started a clinical trial with the objective of comparing the effectiveness of 1000 mg of ferric carboxymaltose with fractionated iron sucrose 200 g/48 h for the treatment of postoperative anaemia, by measuring the change of haemoglobin (Hb) levels from postoperative day (POD) 1 to POD 30.

**Methods:**

We designed an open label randomised controlled trial to compare two postoperative IVI treatment regimens. Patients aged > 18 years undergoing CRC surgery, with Hb < 11 g/dL on POD 1 are randomly assigned to receive either 1000 mg of ferric carboxymaltose (single dose) or 200 g/48 h of iron sucrose. The main study endpoint will be the change from POD 1 to POD 30 in Hb levels and the key secondary endpoint the percentage of patients with Hb levels ≥ 13 g/dL at POD 30. Other secondary endpoints include: changes in iron metabolism parameters (Fe, ferritin, transferrin, % saturated trasferrin) at POD 30; total doses of iron received; number of postoperative transfusions; compliance with oral iron treatment; number of medical and surgical complications; adverse reactions reported by the patient; use of health resources after surgery; and changes in quality of life (QoL). It has been estimated that a sample of 48 patients per group will allow detecting a difference of 0.75 g/dL in Hb in the change in Hb levels from POD 1 to POD 30.

**Discussion:**

The results of this study will confirm if the single dose of 1000 mg ferric carboxymaltose should be preferred in front of the fractionated doses and in which type of patients this regimen should be used preferably.

**Trial registration:**

European Union Clinical Trials Register, EudraCT 2015-001005-13. Registered on 6 January 2015.

**Electronic supplementary material:**

The online version of this article (10.1186/s13063-018-3125-2) contains supplementary material, which is available to authorized users.

## Background

Patients with colorectal cancer (CRC) often present with associated anaemia which can be attributed mainly to iron deficiency due to blood loss and to the inflammation inherent to neoplastic processes [1]. Anaemia is usually present at the time of diagnosis and is aggravated during the postoperative period due to blood loss during the surgery process. On the other hand, it has been well established that preoperative anaemia increases the risk of allogeneic blood transfusions during the perioperative period and that transfusions are associated with an increase in postoperative infections, a higher rate of tumour recurrences and an increase in five-year mortality [1–3]. This is why alternative strategies to optimise patients with preoperative anaemia have been implemented to avoid transfusions. Intravenous iron (IVI) has been shown as an efficacious alternative to transfusion [4–7] and is now recognised as more effective than the oral route in this setting. The main reasons why oral iron has been superseded by IVI are malabsorption due to the inflammation associated to the neoplastic process and the poor compliance due to gastric intolerance [8–10].

The optimisation of CRC cancer patients with anaemia before undergoing surgery was well established in our centre a decade ago, after a multidisciplinary team implemented a diagnostic track for this type of patients [11]. According to the diagnostic track, all patients presenting with anaemia at the time of diagnosis should be treated before surgery with IVI. An expert consensus of different Spanish scientific societies addresses the management of anaemia in surgical procedures with medium-high bleeding, such as CRC surgery, and advises that it would be desirable to reach haemoglobin (Hb) levels > 13 g/dL before surgery in order to minimise the risk of transfusion and quickly correct the anaemia [10, 12]. However, although this document also recommends treatment of postoperative anaemia with IVI with the aim of minimising transfusions and complications, there is no robust evidence to endorse this recommendation.

Titos et al. describe a retrospective study [13] where treatment with IVI after CRC surgery did not affect the needs of transfusion nor did it significantly increase Hb levels. However, it has to be taken into account that the sample size was small and that patients were only studied up to the moment they were discharged; thus, doses received could have been insufficient to induce a significant increase in Hb levels.

Currently, the trend in CRC surgery is to initiate food intake and mobilisation as soon as possible to achieve a prompt functional recovery of the patient and eventually shorten hospital stay. In this situation, the treatment of postoperative anaemia in patients with CRC should be done early and preferably by IV route, as the patient is discharged very soon after surgery.

In our hospital, there is an established protocol for the treatment of postoperative anaemia when it is detected in these patients, aiming at the normalization of Hb levels. This protocol establishes that all patients intervened for CRC, independent of the Hb levels and iron status, must receive during the early postoperative period 200 mg of IVI to substitute the intraoperative loss of blood. On postoperative day (POD) 1, patients must have blood tests with a haemogram. If Hb is < 11 g/dL patients will receive one dose of 1000 mg of ferric carboxymaltose. If Hb is between 11 and 13 g/dL, patients receive 200 mg/48 h of iron sucrose during hospital stay until iron deficit is solved (calculated according to Ganzoni Formula) and after hospital discharge, they will be prescribed oral iron in case the target doses have not been completed. To implement this protocol, two formulations are available at our institution: ferric carboxymaltose 500 mg strength and iron sucrose 100 mg strength.

Despite the advantages of the single 1000-mg doses of ferric carboxymaltose administration, that would guarantee that patients receive the iron load needed before hospital discharge. In a recent retrospective study, we observed an underuse of the 1000-mg single dose even in patients with low Hb levels [14]. On the one hand, the difference in cost compared to fractionated formulations of iron sucrose may account for this. However, the lack of acceptance of such protocols may also be related to the limited evidence perceived by surgeons of the benefit of the 1000-mg doses over the fractionated doses. In fact, there is a lack of information of which is the clinical impact of the two different approaches. Thus, we have considered that comparing both schemes would give an answer to a relevant clinical question that may impact clinical practice in the future. Our hypothesis is that treatments with a single dose of ferric carboxymaltose in patients who present with Hb levels < 11 g/dL at POD 1 will be more effective in achieving normal Hb levels at POD 30.

## Participants and methods

### Study design and interventions

**Fig. 1:**
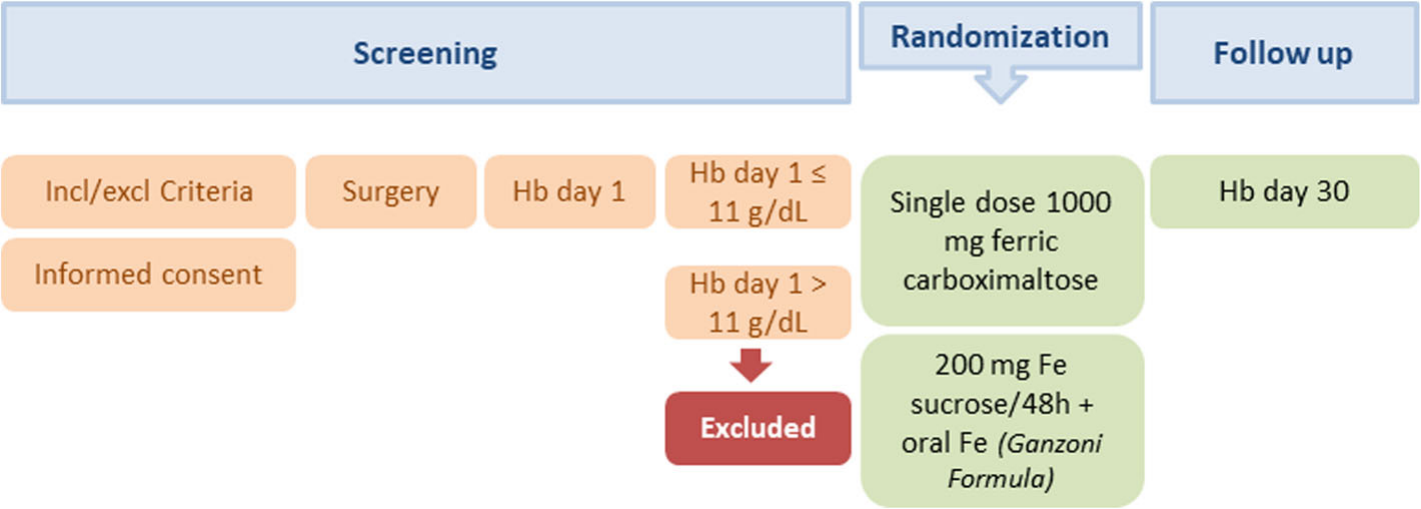
Study design. Ganzoni Formula: Total iron dose (mg iron) = body weight (kg) × (target – actual Hb) (g/dL) × 2.4 + iron for iron stores (mg iron)*. *Iron stores: body weight < 35 kg = 15 mg/kg body weight; body weight > 35 kg = 500 mg

This is a single-centre, open-label randomised controlled trial to compare the effectiveness of two different IVI regimens to treat postoperative anaemia in patients undergoing CRC surgery. During the visit before surgery with the anaesthesiologist, patients meeting the eligibility criteria will be informed and those willing to participate will sign the informed consent form. On POD 1, Hb levels will be determined and those patients presenting with levels < 11 g/dL will be randomly assigned to one of the following study treatments: (1) single dose of 1000 mg of ferric carboxymaltose (Ferinject®, Vifor, France) intravenously in a 15-min infusion; or (2) repeated doses of 200 mg of iron sucrose (different brand names) every 48 h from POD1 up to hospital discharge or up to the total dose equivalent to iron deficit calculated according to the Ganzoni formula, whichever occurs first. Patients who are discharged before achieving the calculated doses will be prescribed oral iron up to POD 30. Patients with Hb levels ≥ 11 g/dL on POD 1 will not be included in the study and will follow the treatment according to hospital protocols. After discharge, patients will be followed up to POD 30 (Fig. 1).

### Study population

Patients aged > 18 years undergoing programmed CRC surgery, with Hb levels < 11 g/dL on POD 1 and who have signed the informed consent form (ICF) are included in the study. Patients who present with Hb levels ≥ 11 g/dL on POD 1, with anaesthetic risk ASA 4, with Claviens classification of surgical complications grade 4, pregnant or lactating women, and patients with previous known intolerance/adverse reactions or with contraindications to IVI are excluded.

### Randomisation

A computer-generated block randomisation (block size 8) has been created with WinPepi etcetera module version 3.26. Randomisation has been stratified by Hb level on day 1 (Hb < 10 g/dL or Hb ≥ 10 g/dL). Sealed envelopes have been created for each individual patient. Pre-screened patients are randomly assigned to one of the two study treatments on POD 1 and after knowing the results of the blood tests for Hb levels.

### Study procedures

**Fig. 2:**
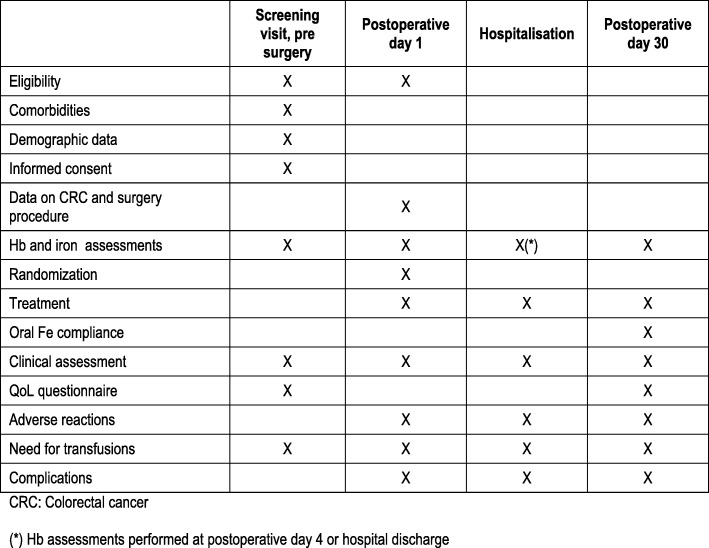
Schedule of enrolment, interventions and assessments

During the preoperative anaesthesiology visit, patients meeting eligibility criteria will be informed and those willing to participate in the study will sign the ICF. During this visit, and after signing the ICF, patients will complete a quality-of-life questionnaire (QL-C30). On POD 1, and after checking eligibility (Hb levels < 11 g/dL), treatment will be randomly assigned to one of the two treatment arms. After hospital discharge, patients will come for a visit on POD 30 when blood tests assessing iron metabolism and Hb will be performed and patients will be asked to complete the QL-C30 questionnaire. Any additional blood tests assessing Hb levels and iron metabolism performed from patient diagnosis to end of trial (i.e. at diagnosis, immediately before surgery and on POD 4 or hospital discharge) will be recorded. When patients are hospitalised, regular checks for adverse events or any complication are performed. Iron treatment will be discontinued in patients admitted in the intensive care unit on POD 1 due to organic fail or presenting Claviens grade 4 complications. Whenever possible, these patients will be followed up to POD 30 (Fig. 2).

### Study endpoints

The main study endpoint will be the change in Hb levels from POD 1 to POD 30. The percentage of patients with Hb levels ≥ 13 g/dL at POD 30 will be considered as a key secondary endpoint. Other secondary endpoints include: changes in iron metabolism parameters (Fe, ferritin, transferrin, % saturated trasferrin) at POD 30; total doses of iron received up to POD 30; number of postoperative transfusions; percentage of patients not compliant with oral iron treatment; number of medical and surgical complications; adverse reactions reported by the patient; and use of health resources after surgery: number of admissions, length of hospital stay; changes in quality of life (QoL). Potential adverse reactions of IVI are headache, dizziness, nausea, abdominal pain, constipation, diarrhoea, exanthema, hypophosphatemia and increases in alanine-aminotransferase. Hypersensitivity and injection site reactions are rare. Oral iron treatment is associated with gastric intolerance and constipation.

### Data collection and management

Study data will be collected in paper case report forms (CRF) designed for the study. Risk adjusted monitoring will be performed by personnel independent from the investigator team, consisting on checking all informed consent forms and completeness of all CRFs and source data verification for a random sample of patients. Data will be entered in the study database by members of the study team. Patients will be identified by a numerical code and no personal information will be included in the paper CRF and the database.

### Statistical methods

#### Sample size

Our hypothesis is that the difference in change in Hb levels (primary endpoint) between the two studied regimens will be 0.75 g/dL. With a two-sided risk of 5% and a power of 80%, and assuming a common standard deviation of 1.3, a total of 48 evaluable patients per group are to be included.

#### Populations of analysis

For safety analysis, all patients who have received at least one dose of IVI will be considered, in the group of treatment they actually received.

The intention to treat (ITT) population will consist of all randomised patients with at least one Hb measurement after POD 1. Patients with missing POD 30 assessments (main endpoint) will be assigned the last value available after POD1 (if any assessment is available). A modified intention to treat population (mITT) will consider all randomised patients with Hb assessments available at POD 30 irrespective of whether they have adhered to the study protocol procedures.

The per-protocol (PP) population will consist of patients in the mITT populations who have received treatment according to the study protocol and for whom an 80% adherence to oral iron is reported and with no relevant protocol deviation that may affect main efficacy assessments (i.e. transfusion).

Although the main efficacy analysis will be performed on the PP population, the other two populations will be used for a sensitivity analysis for the main endpoint.

#### Descriptive statistics

Patient characteristics will be described by treatment group and for the entire sample of patients. Quantitative variables will be presented as means with standard deviations or in cases of skewed distribution as medians and interquartile ranges. Qualitative data will be presented as absolute and relative frequencies.

#### Inferential statistics

Differences in the main variable (change in Hb levels from POD 1 to POD 30) between the treatment groups will be tested by means of an analysis of covariance (ANCOVA) using Hb at POD 1 as covariable. Chi-square test will be used for the key secondary variable, percentage of patients with Hb levels at POD 30 ≥ 13 g/dL, as well as for other categorical variables. For categorical ordinal variables the ANOVA or Kruskall–Wallis test will be used. Continuous variables will be analysed by means of T-test or the corresponding non-parametric tests. A subgroup analysis for the stratification variable (Hb < 10 g/dL and Hb ≥ 10 g/dL) is planned.

An exploratory multivariate analysis will be performed to identify characteristics of patients associated to larger changes in Hb levels and to identify subgroups of patients that may benefit most of the single IVI dose.

### Feasibility

Despite the large number of elective CRC surgeries performed in our hospital (200 CRC surgeries are performed each year on average), recruitment is limited by the fact that only patients with Hb levels < 11 g/dL can be included. Recruitment started in September 2015 and is planned to finish in September 2018.

### Ethics

Written informed consent will be obtained from each participant before any trial-related procedures are carried out. This trial is being conducted in accordance with the Declaration of Helsinki and good clinical practice principles. The study protocol (Version 2, April 2015) was approved by the Research Ethics Committee of *Corporació Parc Taulí* in April 2015 and by the Spanish Medicines Agency (*Agencia Española de Medicamentos y Productos Sanitarios*) in July 2015. The study is registered in the EU Clinical Trials Register (EudraCT: 2015–001005-13). The present study protocol has been written according to the Recommendations for Interventional Trials (SPIRIT) 2013 statement for reporting a clinical trial protocol [15]. The SPIRIT checklist is provided in Additional file 1.

## Discussion

We have designed a randomised controlled study to compare two IVI regimens to treat CRC postoperative anaemia in terms of change in Hb levels from POD 1 to POD 30. Other variables, such as percentage of patients presenting with normal Hb levels at POD 30, changes in iron metabolism parameters, safety of both IVI regimens and complications and QoL, have been considered.

According to our procedures, patients with preoperative anaemia follow an optimisation treatment with the aim of reaching adequate levels before surgery and thus minimising postoperative anaemia and the need for transfusions. In addition, in our hospital, all patients receive 200 mg of iron sucrose right after surgery to account for surgical blood loses. There are no clear recommendations on the use and efficacy of postoperative iron treatment, especially in patients who have already received IVI before surgery. In these cases, the risk of iron overload has not been demonstrated. However, there are a large number of patients who arrive at the postoperative period with Hb levels lower than desired [16, 17]. This may lead to complications, a longer hospital stay and eventually to a slower recovery.

Currently, CRC patients without surgical complications are discharged after 3–4 days. This implies that, for some patients, the treatment with fractionated IVI cannot be completed to the calculated doses and thus they are prescribed oral iron after discharge. However, the absorption of oral iron in this context is known to be minimal [1, 8–10, 12] and, together with the low compliance expected due to mild but unpleasant side effects, patients end up receiving doses lower than required. To overcome these problems, a single dose of 1000 mg is an option for patients with lower Hb levels. However, the high cost of this formulation from a commercially available brand results in an underuse of this regimen in this setting as evidenced in a previous study [14]. Thus, it is of importance to assess whether, in patients with Hb levels < 11 g/dL, treatment with single doses of IVI may lead to larger increases in Hb levels and if this benefit depends on POD 1 Hb levels or other characteristics of patients, in order to make evidence-based decisions.

One of the main limitations of the study is the lack of blinding. Masking of the different administration schedules would need a double dummy strategy which would modify the pragmatic approach of the study. Additionally, the manufacturing of placebo and the masking procedures are a hurdle for an investigator-initiated study, needing the logistic and economic support, in most cases, of the marketing authorisation holder, a situation which is not desirable for an independent trial. On the other hand, the main endpoint is based on an analytical parameter and thus it is less prone to be affected by observation bias.

In addition, while we consider that studying the QoL is a relevant endpoint, we foresee that changes in QoL will be difficult to interpret due to the health condition of patients before surgery.

In a previous observational study [14], we observed that patients treated with postoperative IVI, increased Hb levels in about 2 points at POD 30. Despite these increases, a considerable number of patients do not reach normal levels. Thus, we expect changes of this magnitude and expect them to be larger with the single dose of ferric carboxymaltose than with the fractionated doses regimen with iron sucrose. We also hypothesise that patients with lower Hb levels will benefit more from the single-dose regimen.

The study is being performed in only one centre and, in most cases, the principal investigator, an anaesthetist, is responsible for the inclusion, randomisation and treatment of the patient, ensuring uniformity of procedures. Additionally, adapted risk monitoring has been implemented to ensure that the protocol is performed according to GCP criteria and to ensure the quality of data.

The results of this study will confirm whether the single dose of 1000 mg of ferric carboxymaltose should be preferred ahead of the fractionated doses and in which type of patients this regimen should preferably be used.

## Trial status

The trial is ongoing. At the time of finishing this manuscript, 122 patients have been screened and signed informed consent. Of those, 75 have been randomised and 47 were not eligible, mainly due to Hb levels on POD1 ≥ 11 g/dL.

## Additional file

Additional file 1: SPIRIT 2013 Checklist: Recommended items to address in a clinical trial protocol and related documents*. (DOC 122 kb)

## Abbreviations

ASA: American Society of Anaesthesiologists physical status classification
CRC: Colorectal cancer
CRF: Case report form
GCP: Good Clinical Practice
Hb: Haemoglobin
ICF: Informed consent form
ITT: Intention to treat
IVI: Intravenous iron
mITT: Modified intention to treat
POD: Postoperative day
PP: Per protocol
QoL: Quality of life
